# High glucose induces upregulation of scavenger receptors and promotes maturation of dendritic cells

**DOI:** 10.1186/1475-2840-12-80

**Published:** 2013-05-29

**Authors:** Hao Lu, Kang Yao, Dong Huang, Aijun Sun, Yunzeng Zou, Juying Qian, Junbo Ge

**Affiliations:** 1 Department of Cardiology, Zhongshan Hospital, Fudan University, Shanghai Institute of Cardiovascular Diseases, 180 Fenglin Road, Shanghai China 200032

**Keywords:** Dendritic cell, Scavenger receptors, Type 2 diabetes, Atherosclerosis, Immune response

## Abstract

**Background:**

Both hyperglycaemia and dendritic cells (DCs) play causative roles in atherosclerosis. However, whether they interact in atherosclerosis remains uncertain. Therefore, we examined whether high glucose could regulate the expression of scavenger receptors responsible for oxidised low-density lipoprotein (oxLDL) uptake in DCs, a critical step in atherogenesis. In addition, we investigated the impact of glucose on DC maturation regarding changes in phenotype and cytokine secretion.

**Methods:**

Immature DCs were cultured with different concentrations of glucose (5.5 mmol/L, 15 mmol/L, 30 mmol/L) in the absence or presence of N-acetylcysteine (NAC), SB203580 or Bay11-7082 for 24 hours. We used 30 mmol/L mannitol as a high-osmolarity control treatment. The expression of the scavenger receptors SR-A, CD36 and LOX-1 was determined by real-time PCR and western blot analysis. Furthermore, DCs were incubated with DiI-labelled oxLDL. The DiI-oxLDL-incorporated fraction was investigated by flow cytometry analysis. The intracellular production of ROS in DCs was measured by dichlorodihydrofluorescein (DCF) fluorescence using confocal microscopy. Finally, flow cytometry analysis was used to investigate immunophenotypic protein expression (CD83 and CD86). Supernatant cytokine measurements were used for immune function assays.

**Results:**

The incubation of DCs with glucose enhanced, in a dose-dependent manner, the gene and protein expression of SR-A, CD36 and LOX-1. This effect was partially abolished by NAC, SB203580 and Bay11-7082. Incubation of DCs with mannitol (30 mmol/L) did not enhance these scavenger receptors’ expression. High glucose upregulated the production of ROS and expression of p38 MAPK in DCs. NAC partially reversed p38 MAPK upregulation. High glucose increased the oxLDL-uptake capacity of DCs. Blockage of the scavenger receptors SR-A and CD36 reduced oxLDL uptake, but blockage of LOX-1 did not. Furthermore, high-glucose (15 mmol/L or 30 mmol/L) treatment increased CD86 and CD83 in DCs. High glucose also increased IL-6 and IL-12 secretion and decreased IL-10 secretion.

**Conclusion:**

High glucose can increase the expression of the scavenger receptors SR-A, CD36 and LOX-1, which can increase the oxLDL-uptake capacity of DCs. High glucose induces a proinflammatory cytokine profile in human DCs, leading to DC maturation. These results support the hypothesis that atherosclerosis is aggravated by hyperglycaemia-induced DC activation and oxLDL uptake.

## Introduction

Cardiovascular complications remain the leading cause of mortality in adults with diabetes. Hyperglycaemia is the hallmark of diabetes and is a major independent risk factor for diabetic macrovascular disease, playing a key pathogenic role in the development of diabetes-associated atherosclerosis [1-3]. However, a clear causative role for hyperglycaemia has not been established. The major biochemical pathways of hyperglycaemic vascular damage and the hyperglycaemia-induced activation of nuclear transcription factor-kappa B (NF-κB) result from a single common mechanism: overproduction of reactive oxygen species (ROS) [4]. The overproduction of ROS in poorly controlled diabetes could contribute to endothelial and vascular dysfunction, leading to atherosclerosis. Recent studies in animal models indicate that glucose may play a role in diabetes-accelerated atherosclerosis by promoting pro-inflammatory responses in monocytes and macrophages [5].

Atherosclerosis is a chronic inflammatory vessel disease characterised by early endothelial dysfunction. In response to endothelial activation, monocytes and T-cells mediate the progression of atherosclerosis. Dendritic cells (DCs) are a specific type of leukocyte that alert the immune system to the presence of antigens, infections and inflammatory mediators. They play a central role in the initiation of both innate and adaptive immune responses [6]. In recent years, the impact of DCs on the initiation and progression of atherosclerosis has been evaluated [7]. DCs home to the vessel wall and recognise foreign and autoantigens (viral and bacterial antigens, oxidised LDL (oxLDL), HSP 60/65) [8,9]. oxLDL uptake might result in enhanced presentation of lipid and peptide antigens to NKT and T-cells and further stimulate vascular inflammation and adhesion of monocytes in the atherosclerotic plaque^9^. Scavenger receptors, which are expressed by macrophages and monocyte-derived DCs, are major receptors for oxLDL. The stimulation of DCs by oxLDL through binding to scavenger receptors leads to their activation and can be accompanied by enhanced cytokine production [10]. Furthermore, although lipid uptake and foam cell formation in the artery have been attributed mostly to macrophages, recent studies have demonstrated that DCs in the subendothelial space of the aorta can also efficiently accumulate lipids and differentiate into foam cells, thereby contributing to the initiation and further progression of atherosclerosis [11]. SR-A, CD36 and LOX-1 are essential scavenger receptors for uptake of oxLDL and foam cell formation.

Elevated glucose can increase the expression of scavenger receptors CD36 [12], SR-A [13] and LOX-1 [14] in macrophages, thereby contributing to diabetes and its related disease atherosclerosis. However, to the best of our knowledge, little is known about how glucose affects the expression of scavenger receptors in DCs. Therefore, we examined whether high glucose regulates scavenger receptor expression in DCs, focusing on CD36, SR-A and LOX-1, and whether glucose modulates the maturation processes of DCs. In addition, we analysed the impact of glucose on ROS production and the NF-κB pathway in DCs.

## Methods

### Materials

Human CD14^+^ immunomagnetic microbeads were obtained from Miltenyi Biotech GmbH (Bergisch-Gladbach, Germany). Recombinant human granulocyte/macrophage colony-stimulating factor (rhGM-CSF), recombinant human interleukin-4 (rhIL-4), enzyme-linked immunosorbent assay (ELISA) kits for IL-6, IL-10, IL-12p70 and tumour necrosis factor-α (TNF-α) were from R&D Systems. (United States).Histopaque-1077, D-glucose, N-acetyl cysteine (NAC) and FITC-dextran were from Sigma. Trizol reagent was from Invitrogen (United States). The reverse-transcription system and GoTaq qPCR Master Mix were from Promega (United States). The human T-cell recovery immunocolumn kit was from Cedarlane Laboratories Ltd. Goat anti-human SR-A polyclonal antibody and mouse anti-human CD36 monoclonal antibody were from Santa Cruz Biotechnology. Rabbit anti-human LOX-1 polyclonal antibody was from Abcam. Mouse anti-human CD83-FITC, CD86-FITC and HLA-DR-FITC antibodies were from BD Pharmingen. DiI-oxLDL was purchased from Peking Union-Biology Co. Ltd. DCFH-DA was purchased from Molecular Probes (United States). Bay11-7082 was purchased from Merck (United States).

### Generation of monocyte-derived dendritic cells

Peripheral blood mononuclear cells (PBMCs) were obtained from healthy volunteers. Briefly, blood was diluted 1:2 in PBS layered over Histopaque 1077 and centrifuged for 30 min at 2000 rpm at room temperature. The interface was recovered and washed three times in PBS. CD14^+^ PBMCs were purified by using CD14^+^ immunomagnetic micro beads and incubated in RPMI-1640 medium supplemented with GM-CSF (100 ng/mL) and IL-4 (50 ng/mL) in six-well tissue culture plates at 37°C and an atmosphere of 5% CO_2_. The medium was replaced every 2 days. On day six, cells were exposed to glucose at various concentrations (5.5 mmol/L, 15 mmol/L, 30 mmol/L) for a further 24 h. In control experiments, mannitol was added to the medium to bring total osmolality to a value equivalent to 30 mmol/L glucose (high glucose). In some experiments, 10 mmol/L NAC, 10 μmol/L SB203580 or 10 μmol/L Bay11-7082 was added simultaneously with high glucose.

### Real-time PCR

The mRNA expression of the different scavenger receptors (SR-A, CD36 and LOX-1) in DCs was analysed using real-time quantitative reverse transcription–polymerase chain reaction (RT-PCR). Total RNA was isolated and treated with DNase using a Trizol Reagent kit according to the manufacturer’s instructions. Five micrograms of the total RNA sample was reverse-transcribed using oligo-dT and SuperScript III. SR-A was amplified using the sense primer 5′-TCCTCGTGTTTGCAGTTCTC-3′ and antisense primer 5′-GCAATTCTTCGTTTCCCACT-3′. CD36 was amplified using the sense primer 5′-CGCTGAGGACAACACAGTCT-3′ and antisense primer 5′-GTTGTCAGCCTCTGTTCCAA-3′. LOX-1 was amplified using the sense primer 5′-GGGCTCATTTAACTGGGAAA-3′ and antisense primer 5′-GAAATTGCTTGCTGGATGAA-3′. Quantitative PCR using SYBR Green reagent was performed on the ABI 7500 Real-time PCR System (Applied Biosystems, United States). Gene expression was analysed by the system software, and the copies of each mRNA molecule were determined by the standard curve method.

### Western blotting

After treatment with glucose, DCs were washed with ice-cold PBS and were then lysed in cold cell lysis buffer. Protein concentration was measured using a BCA Protein Assay Reagent kit (Beyotime Institute of Biotechnology). Protein extracts (20 μg) were separated by 7.5% SDS-PAGE, transferred to a nitrocellulose membrane by electrotransfer (200 V for 30–60 min) and blocked with 5% nonfat milk for 1 h at room temperature. Antigen–antibody complexes were detected using an appropriate HRP-labelled secondary antibody with the ECL detection system (Thermo Fisher Scientific, Rockford, IL, USA) according to the manufacturer’s protocol. The resulting bands were analysed densitometrically using ImageQuant software (Molecular Dynamics, Sunnyvale, CA, USA). All values were normalised to the tubulin loading control.

### oxLDL uptake

After treatment with 5.5 mmol/L or 30 mmol/L glucose for 24 h, DCs were incubated with DiI-labelled oxLDL (10 μg/ml) for 60 min at 37°C. Then, cells were harvested and washed three times at 4°C. The DiI-oxLDL-incorporated fraction was quantified by flow cytometry analysis.

### Blocking oxLDL uptake

DCs were incubated with DiI-oxLDL (10 μg/ml) for 4 h at 4°C with or without anti-SR-A (Biozol, USA), anti-CD36 (Abcam, USA) and anti-LOX-1 (R&D Systems, USA) neutralizing antibodies. The endocytosed DiI-oxLDL fraction was analysed using flow cytometry analysis. To rule out nonspecific blocking, control IgG antibodies were used.

### Measurement of intracellular reactive oxygen species

After 24 h incubation with high-glucose medium, the ROS probe dye DCFH-DA (10 μmol/L) was added. Intracellular production of ROS in DCs was measured by dichlorodihydrofluorescein (DCF) fluorescence using confocal laser-scanning microscopy (Zeiss Axiovert 135 microscope, Leica TCs-SP2 confocal attachment, laser excitation 488 nm, emission long-pass LP515-nm filter set). Images of all samples were collected by single rapid scans under identical parameters, such as contrast and brightness. Four groups of 20 cells for each sample were randomly selected from the image, and fluorescence intensity was measured. The relative fluorescence intensities are the average values of all experiments.

### Flow cytometric measurement

On culture day 7, cells were washed and resuspended in ice-cold PBS containing 5% foetal bovine serum to prevent nonspecific binding, then incubated with FITC-conjugated mAbs (CD83-FITC, CD86-FITC) for 30 minutes at 4°C. Then, the cells were analysed using a flow cytometer (BD Biosciences) and Cell Quest software. Cells stained with the appropriate isotype-matched Ig were used as negative controls.

### DC cytokine secretion

The supernatants of human DC culture (±24 h incubation with different concentrations of glucose) were harvested and stored at −80°C until they were analysed for the presence of IL-6, IL-10, IL-12p70 and TNF-α. These cytokines were determined using cytokine-specific ELISA kits according to the manufacturer’s instructions.

### Statistical analysis

Data are presented as the mean ± standard deviation of the mean (SDM). Group mean values were compared with 1-way analysis of variance followed by post hoc tests using the Tukey procedure for pairwise comparisons. All in vitro experiments were repeated at least three times with different cells. All statistical analyses were performed with SPSS for Windows 13.0. P values less than 0.05 were considered statistically significant.

## Results

### Effects of glucose on SR-A, CD36 and LOX-1 mRNA expression in DCs

**Figure 1:**
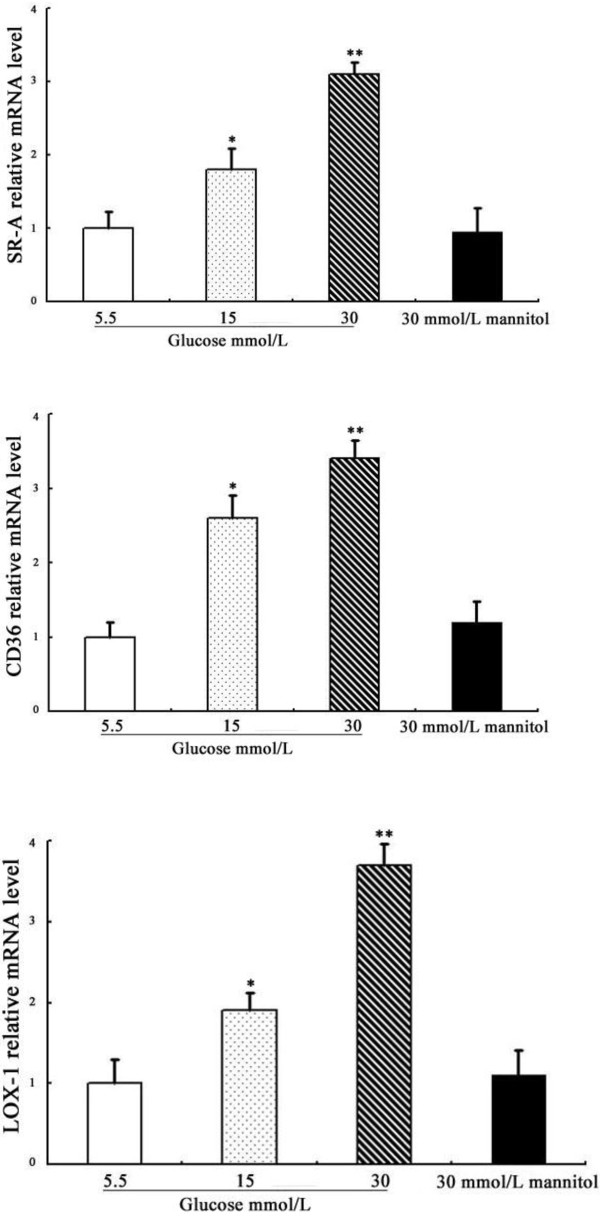
**Effects of glucose on the mRNA expression of SR**-**A, CD36 and LOX-1 in DCs.** SR-A, CD36 and LOX-1 mRNAs were analysed by real-time quantitative RT-PCR. Representative examples of the dose-dependent expression of SR-A, CD36 and LOX-1 on DCs. Mean ± SEM. n = 3. *p < 0.05 vs. 5.5 mmol/L glucose, **p < 0.01 vs. 5.5 mmol/L glucose.

We first studied the effects of different concentrations of glucose on the expression of the SR-A, CD36 and LOX-1 genes in DCs. As shown in Figure 1, the stimulatory effects of glucose on SR-A, CD36 and LOX-1 mRNA expression were dose-dependent, with the maximal effects occurring at 30 mmol/L glucose. The incubation of DCs with mannitol (30 mmol/L) did not enhance these scavenger receptors’ mRNA expression, suggesting that the upregulation of scavenger receptors by high glucose is not due to the osmotic effect.

### Effects of glucose on SR-A, CD36 and LOX-1 protein expression in DCs

**Figure 2:**
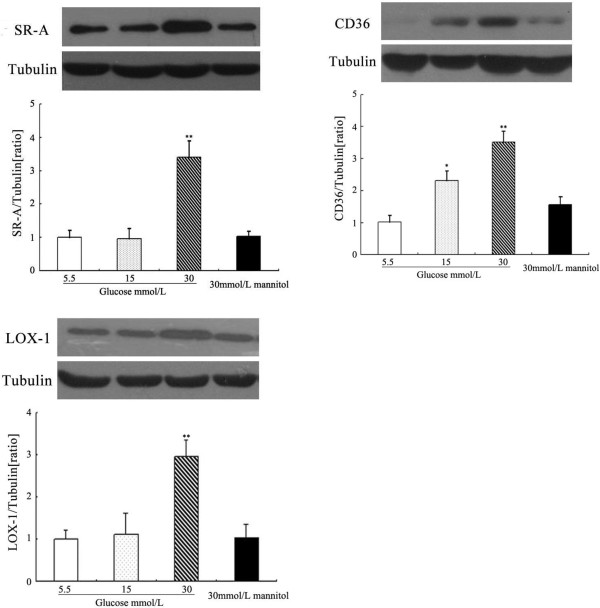
**Effects of glucose on SR-A, CD36 and LOX-1 protein expression in DCs.** SR-A, CD36 and LOX-1 proteins were analysed by western blotting. Densitometric analysis showed significantly increased expression of SR-A, CD36 and LOX-1 after prestimulation by 30 mmol/L glucose. Mean ± SEM. n = 3. *p < 0.05 vs. 5.5 mmol/L glucose, **p < 0.01 vs. 5.5 mmol/L glucose.

The changes observed at the protein level were similar to those described for mRNA. Incubating DCs for 24 hours with increasing glucose concentrations (5.5 to 30 mmol/L) enhanced, in a dose-dependent manner, SR-A, CD36 and LOX-1 protein expression. SR-A, CD36 and LOX-1 protein levels normalised to the level of tubulin are illustrated in Figure 2. No stimulatory effect of mannitol (30 mmol/L) on SR-A, CD36 or LOX-1 protein expression was observed (Figure 2).

### oxLDL uptake and blockage of scavenger receptors

**Figure 3:**
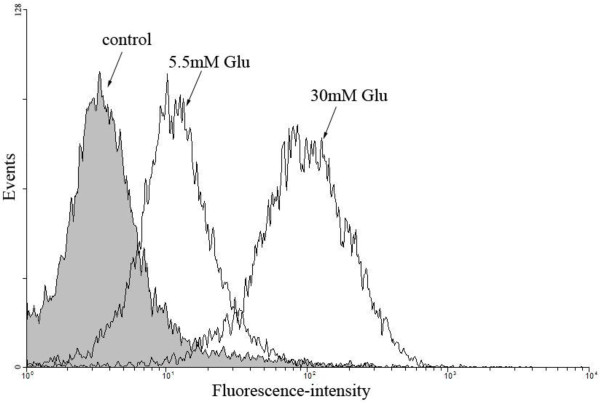
**Effects of glucose on the uptake of DiI-oxLDL by DCs.** Representative example of the effects of 5.5 mM Glu and 30 mM Glu on the uptake of DiI-oxLDL by DCs at 37°C.

As SR-A, CD36 and LOX-1 are high-affinity scavenger receptors for oxLDL, we next examined the effects of glucose on uptake of DiI-labelled oxLDL by DCs. When the cells were treated with 30 mmol/L glucose, an increase of more than twofold in the uptake of oxLDL was observed compared to 5.5 mmol/L glucose-treated cells, as assessed by flow cytometric analysis (Figure 3).

**Figure 4:**
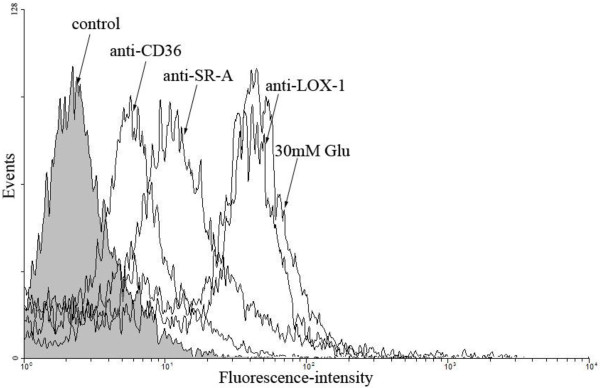
**Blocking the uptake of DiI-oxLDL by DCs.** Representative histograms of blocking DiI-oxLDL uptake using antibodies against SR-A, CD36 and LOX-1 at 4°C. 30 mM Glu: 30 mmol/L glucose.

The uptake of oxLDL was partially inhibited by specific blockage with antibodies against SR-A and CD36. Anti-SR-A reduced oxLDL uptake by 45%, anti-CD36 by 66% (p < 0.01 for all experiments), but anti-LOX-1 did not lead to a reduced oxLDL uptake (p = 0.24) (Figure 4). Furthermore, nonspecific control IgG-antibodies did not affect oxLDL uptake (data not shown).

### Effects of high glucose on the production of ROS in DCs

**Figure 5:**
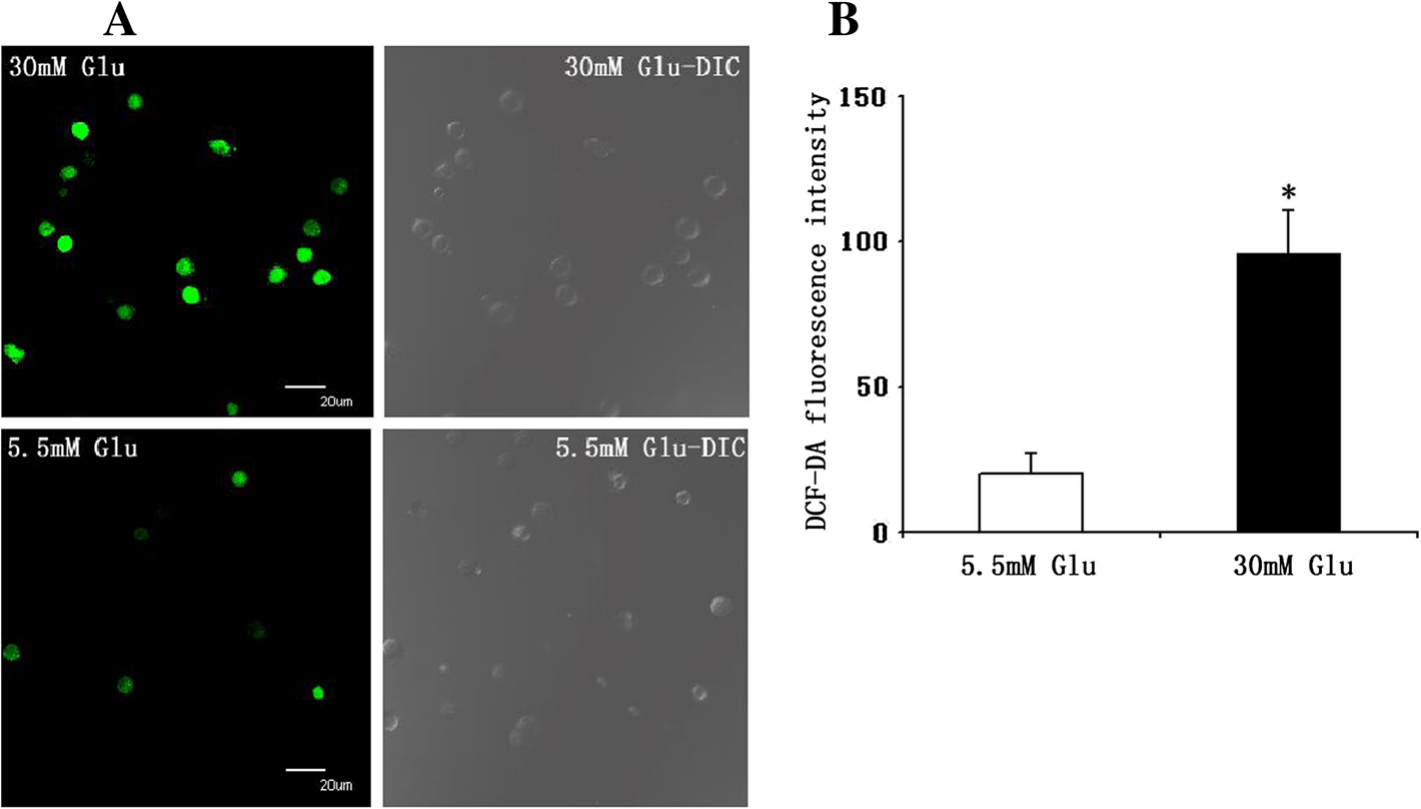
**Effect of high glucose on reactive oxygen species production in DCs.** Reactive oxygen species production was visualised by DCF fluorescence confocal laser microscopy. **A**, Representative microscopic scan. **B**, Quantification of reactive oxygen species production. Data analysis of 4 separate experiments, expressed as relative fluorescence. Mean ± SEM, *p < 0.01 vs. 5.5 mmol/L glucose. DIC: differential interference contrast microscopy.

To examine whether high glucose induces ROS production in cultured human monocyte-derived DCs, we measured intracellular ROS using the redox-sensitive fluorescent dye DCFH-DA. The fluorescence intensity was significantly higher in DCs cultured for 24 hours in high-glucose medium (30 mM glucose) compared with those in normal glucose (5.5 mM glucose). A representative microscopic scan is shown in Figure 5A, and data analysis of three separate experiments is illustrated in Figure 5B. These results suggest that high glucose can induce the formation of ROS in human monocytes-derived DCs.

### Signalling pathways involved in high glucose-induced DCs scavenger receptor expression

**Figure 6:**
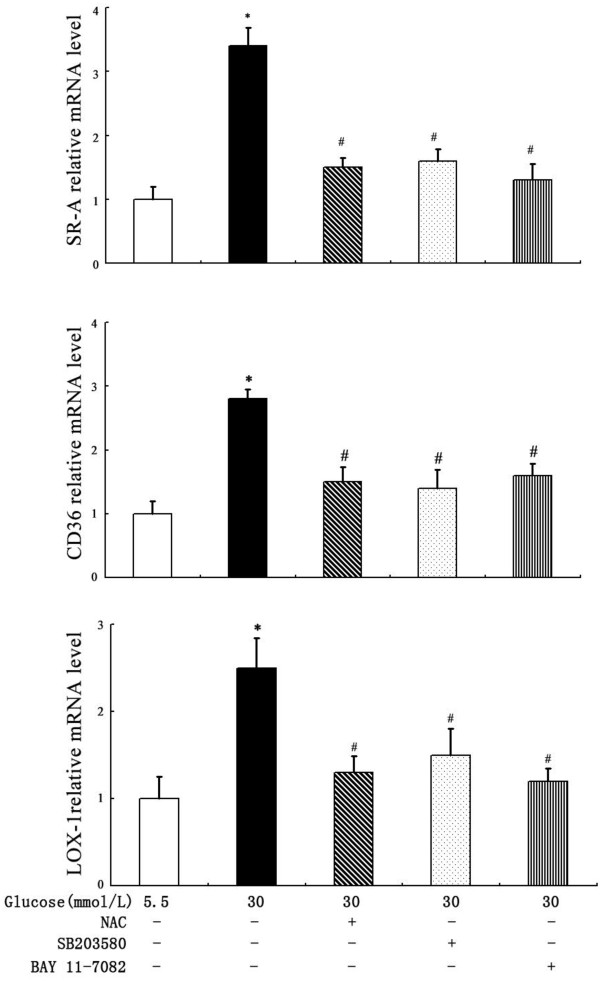
**Effects of p38 MAPK inhibitor, NF-κB inhibitor and NAC on glucose-induced scavenger receptors mRNA levels.** DCs were pretreated for 2 hours with SB203580, NAC or BAY 11–7082 before exposure to high glucose. SR-A, CD36 and LOX-1 mRNAs were quantified by real-time PCR. Mean ± SEM. n = 3. *p < 0.05 vs. 5.5 mmol/L glucose, #p < 0.05 vs. 30 mmol/L glucose.

**Figure 7:**
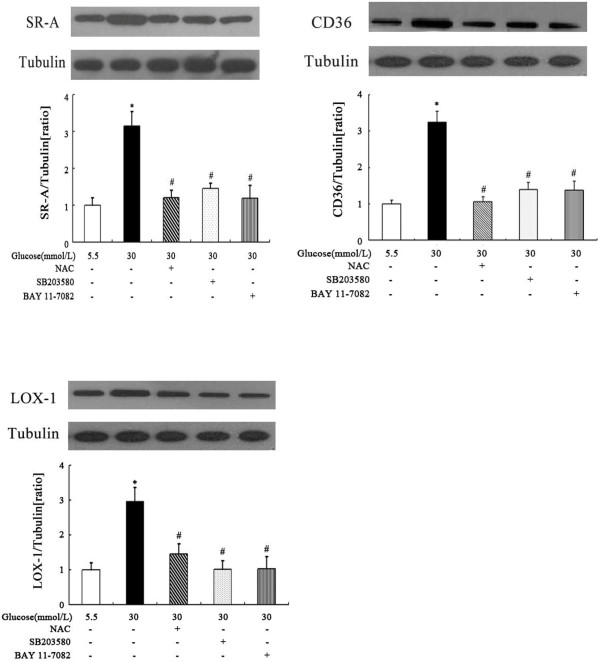
**Effects of p38 MAPK inhibitor, NF-κB inhibitor and NAC on glucose-induced scavenger receptors protein levels.** DCs were pretreated for 2 hours with SB203580, NAC or BAY 11–7082 before exposure to high glucose. SR-A, CD36 and LOX-1 protein levels were quantified by western blotting. Mean ± SEM. n = 3. *p < 0.05 vs. 5.5 mmol/L glucose, #p < 0.05 vs. 30 mmol/L glucose.

To identify the signalling pathways involved in the stimulatory effect of high glucose on scavenger receptor expression, DCs were pretreated for 2 hours with the p38 mitogen-activated protein kinase (MAPK) inhibitor SB203580 (10 μmol/L) or the NF-κB inhibitor BAY 11-7082(10 μmol/L) before exposure to glucose. As shown in Figures 6 and 7, at both the mRNA and protein levels, SB203580 partially abrogated glucose-induced SR-A, CD36 and LOX-1 expression. A similar effect was observed when the cells were pre-incubated with BAY 11–7082 (Figures 6 and 7). Because diabetes and high glucose induce increased oxidative stress, we next determined the role of oxidative stress in the regulation of scavenger receptor expression by glucose. As shown in Figures 6 and 7, the pre-incubation of DCs with the antioxidant N-acetyl-L-cysteine (NAC) (10 mmol/L) prevented the stimulatory effect of high glucose on SR-A, CD36 and LOX-1 expression.

**Figure 8:**
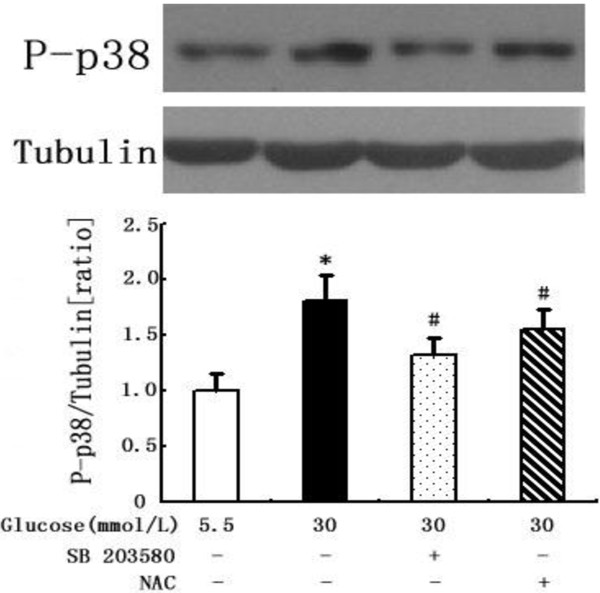
**Inhibitory effects of NAC and SB203580 on p38 MAPK phosphorylation in glucose-stimulated DCs.** DCs were pretreated for 2 hours with SB203580 or NAC before exposure to high glucose. Then, cells were harvested and assayed for the phosphorylation of p38 MAPK by western blots. Mean ± SEM. n = 3. *p < 0.05 vs. 5.5 mmol/L glucose, #p < 0.05 vs. 30 mmol/L glucose.

We next assessed the sequential events leading to glucose-induced p38 MAPK activation. Figure 8 shows that high glucose activated the phosphorylation of p38 MAPK in DCs. The stimulatory effect of high glucose was blocked in the presence of SB203580 (Figure 8). Furthermore, the glucose-induced activation of p38 MAPK was reduced by NAC (Figure 8). Altogether, these results demonstrate that high glucose stimulation activated the p38 MAPK pathway and via ROS generation.

### Effects of glucose on DCs maturation

**Figure 9:**
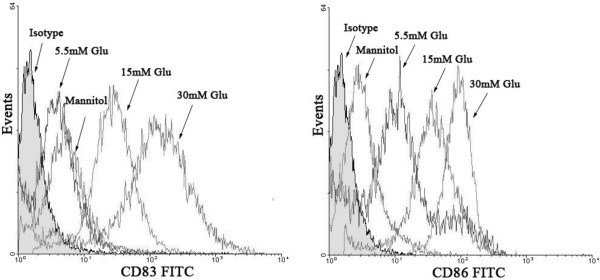
**The immunophenotypic expression of DCs exposed to different concentrations of glucose.** Flow cytometric analysis was performed to estimate cell-surface CD83 and CD86 expression. 5.5 mM Glu: 5.5 mmol/L glucose; 15 mM Glu: 15 mmol/L glucose; 30 mM Glu: 30 mmol/L glucose; mannitol: 30 mmol/L mannitol.

Mature DCs express many co-stimulatory molecules, such as CD83 and CD86. Flow cytometric analysis showed that the expression of CD83 and CD86 in 15 mmol/L and 30 mmol/L glucose-treated DCs increased significantly compared with normal glucose (5.5 mmol/L), and this effect was dose-dependent (Figure 9). These results indicate that high glucose could promote the maturation of DCs. Mannitol at the same osmolality as high glucose did not increase the expression of CD83 or CD86 (Figure 9), suggesting that the acceleration of the maturation process by high glucose is not due to the osmotic effect.

### Effects of glucose on DCs cytokine secretion

**Figure 10:**
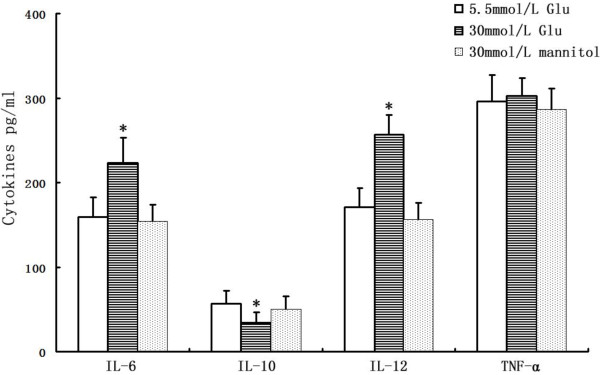
**Cytokine secretion in high glucose-treated DCs.** DCs of different groups were harvested, and the supernatants were collected; IL-6, IL-10, IL-12p70 and TNF-α were determined using commercially available ELISAs. *p < 0.05 vs. 5.5 mmol/L glucose. Mean ± SEM. n = 3.

Mature DCs secrete a wide spectrum of cytokines and chemokines. We examined the influence of glucose (5.6 or 30 mmol/L) on the secretion of IL-6, IL-10, IL-12p70 and TNF-α from DCs by ELISAs. IL-6 (+40%; p < 0.05) and IL-12p70 secretion (+50%; p < 0.05) were increased significantly, and IL-10 was decreased by 38.7% (p < 0.05), whereas TNF-α (+2.2%) was not significantly affected by glucose (Figure 10).

## Discussion

The present study demonstrates that glucose enhanced, in a dose-dependent manner, the expression of the DC scavenger receptors SR-A, CD36 and LOX-1, both at the mRNA and protein levels. This phenomenon was suppressed by a p38 MAPK inhibitor, an NF-κB inhibitor and an antioxidant. Our observation that high glucose enhanced DC ROS generation provides strong evidence for a crucial role of ROS in high glucose-induced scavenger receptor upregulation. We also found that the antioxidant and p38 MAPK inhibitor blocked glucose-induced p38 MAPK activation. Taken together, these results indicate that the increased production of intracellular ROS and the activation of p38 MAPK pathways are initial signalling events in the regulation of scavenger receptor expression by glucose that are required for subsequent activation of NF-κB. We also found that glucose promoted DCs’ ability to take up oxLDL. This capability was partially blocked by neutralising antibodies against CD36 and SR-A, but not LOX-1. Moreover, high glucose induced a proinflammatory cytokine profile in human DCs and triggered DC maturation. To our knowledge, this is the first study showing these important effects of glucose on DCs.

Cardiovascular diseases in patients with type 2 diabetes are a large and increasing health problem. Unfortunately, the exactly mechanisms leading to atherosclerosis in diabetes are only partially understood. Quite recently, Skov V et al. [15] reported that dysregulated gene interactions and pathways in the cells of the arterial wall in diabetes may play important roles in the arterial response to injury and the sequential inflammation. Insulin resistance and hyperglycaemia in type 2 diabetes mellitus are associated with a systemic proinflammatory state that promotes the development of atherosclerosis [16]. Our recent in vitro studies have shown that advanced glycosylation end products (AGEs) [17] and hyperinsulinaemia [18] enhance DC maturation and induce antigen-specific T-cell activation. DCs and oxLDL accumulate in progressive atherosclerotic plaque. In the subendothelial space, DCs phagocytose antigens such as oxLDL. Then, DCs can either accumulate in the arterial wall and/or migrate to draining lymph nodes for antigen presentation [19]. Lipid and oxLDL uptake might result in the enhanced presentation of lipid and peptide antigens to NKT and T-cells. Furthermore, some foam cells can derive from DCs, and the oxLDL uptake capacity of DCs is reflected by the expression of SR-A, CD36 and LOX-1 [10]. The engulfment of lipid by resident intimal DCs and differentiation into foam cells is possibly one of the earliest steps of atherogenesis [20]. In addition, CD36 mediates oxLDL-induced TLR4/TLR6 activation [21]. oxLDL stimulates DCs by binding to CD36 and TLR4, leading to DC activation, which can be accompanied by enhanced cytokine production [22]. The present study shows that high glucose can mediate upregulation of SR-A, CD36 and LOX-1, which could be related to initiation and progression of atherosclerosis in diabetes patients.

High glucose induced an increased endocytic function of DCs, which was determined by a semiquantitative method using a fluorescence-labelled ligand (DiI-OxLDL). This effect was partially but significantly inhibited by an anti-CD36 or anti-SR-A neutralising antibody, suggesting some contribution of CD36 and SR-A to atherosclerosis formation by human DCs under diabetic conditions. However, the anti-LOX-1-neutralising antibody did not show significant inhibition of the endocytosis of DiI-oxLDL by high glucose-treated DCs. The reason may be that LOX-1 is mainly an endothelial-specific scavenger receptor, and although it is also expressed on DCs, macrophages, smooth muscle cells and platelets, its expression level is relatively low in these cell types [23-26]. Therefore, it is likely that LOX-1 is not a major receptor responsible for the uptake of oxLDL in DCs. Further studies are needed to determine the exact contribution of LOX-1 to high glucose-induced upregulation of DCs *in vitro*. The relative contributions of CD36, SR-A and LOX-1 to the diabetes-induced enhancement of foam cell and atherosclerosis formation by DCs has to be clarified by future studies.

The effects of several inhibitors on high glucose-enhanced scavenger receptor expression were examined to determine the signalling pathway between glucose stimulation and scavenger receptor expression. High glucose-enhanced scavenger receptor expression was suppressed by the p38 MAPK inhibitor, NF-κB inhibitor and antioxidant. Hyperglycaemia-induced oxidative stress is implicated in the pathogenesis of chronic diabetic complications. Recent studies [12-14] found that ROS generated by glucose in macrophages may be key intermediates in the regulation of SR-A, CD36 and LOX-1 expression by this metabolic factor. Moreover, Farhangkhoee H et al. [27] found that high glucose-induced upregulation of CD36 may be involved in increased oxidative stress in microvascular endothelial cell. In this study, we found that high glucose can also enhanced ROS generation in DCs. Evidence linking glucose-induced oxidative stress with activation of p38 MAPK in macrophages supports a role for this kinase in the control of scavenger receptor expression by hyperglycaemia [4,28]. In line with these hypotheses, we found that antioxidant and p38 MAPK inhibitor abolished glucose-induced scavenger receptor expression, implicating ROS and kinases as signalling molecules mediating this effect of glucose. Our data also indicate a role for oxidative stress in p38 MAPK activation. Finally, we found that NF-κB inhibitor suppressed glucose-induced scavenger receptor expression. So we propose that high glucose induces DCs to generate ROS and activate p38 MAPK signalling, which leads to the subsequent activation of NF-κB and finally upregulates scavenger receptor expression. But Takaki KF et al. [13] reported that p38 MAPK inhibitor have no effect on high glucose-induced SR-A expression in macrophages, which is different from our present study. We do not know the reason for the discrepancy at present. One possible reason is that Takaki KF et al. used monocyte-derived macrophages whereas we used monocyte-derived DCs. The difference in cell types might cause different results.

In this study, we focussed not only on the impact of glucose on scavenger receptors but also on DC activation. In doing so, we investigated the influence of glucose on DC maturation and cytokine production. CD86 and CD83 are mature markers of DCs. Co-stimulation by the ligand CD86 and its receptor CD28 is required for efficient T-cell stimulation [9]. We found that glucose induced the upregulation of the co-stimulatory receptors CD86 and CD83, which supports the hypothesis that T-cells are activated by DCs in plaque lesions [29]. Consistent with this study, our recent in vivo study [30] also showed that the expression of CD86 was significantly increased in diabetes patients with unstable angina pectoris(UAP), which indicated that the functional status of DCs in diabetic patients with UAP were more mature and activated than none diabetic patients with UAP. The inflammatory activation of DCs is reflected by the differentially expressed cytokine-chemokine spectrum induced by glucose. We discovered that the pro-atherosclerotic chemokines IL-6 and IL-12 are induced by high glucose, while the release of anti-atherosclerotic IL-10 is reduced. IL-10 may have therapeutic potential in various inflammatory diseases, including atherosclerosis, as it can inhibit oxLDL-induced foam cell formation and apoptosis in macrophages and endothelial cells. The inhibitory effect of IL-10 on oxLDL-induced apoptosis was partially dependent on reduced p38 MAPK phosphorylation [31]. IL-6 is a pro-atherogenic cytokine that is locally produced by plaque-infiltrating inflammatory cells [32]. IL-12 enhances the ability of activated human monocytes to oxidise LDL and is able to modify the chemokine production of human vascular smooth muscle cells [33]. IL-12 also seems to be important for both Th1 differentiation within the plaque and for overall T-cell recruitment into the plaque [34]. Considering that DCs are most frequently observed in atherosclerotic lesions enriched with T-cells, the increased expression of costimulatory molecules on DCs and cytokine secretion induced by high glucose suggest that activation of DCs in diabetes patients is an important mediator in immuno-inflammatory process, as DCs are responsible for T-cell activation, and this cellular interaction plays a role in plaque instability and vulnerability, leading to rupture.

## Conclusion

In summary, high glucose can increase the expression of scavenger receptors SR-A, CD36 and LOX-1 on DCs, which can lead to uptake of oxLDL and maturation of DCs. Mature, oxLDL-presenting DCs are essential for priming naive T-cells, thereby inducing an oxLDL-specific T-cell line. (Auto-)Immune-active T-cells are an essential element of the inflammation cascade. This effect is mediated by enhancing ROS generation and signalling via the p38 MAPK and NF-κB pathways. The results of the present study show that one of the mechanisms by which high glucose promotes atherogenesis involves its effects on DCs. These findings may be relevant in understanding the intracellular signalling associated with the pathophysiological processes involved in the growth and destabilisation of atherosclerotic plaques in diabetes patients and provide new pharmacological targets to prevent the development and progression of diabetic complications.

## Abbreviations

DCs: Dendritic cells; NAC: N-acetylcysteine; NF-κB: Nuclear transcription factor-kappa B; ROS: Reactive oxygen species; oxLDL: oxidised LDL; rhGM-CSF: recombinant human granulocyte/macrophage colony-stimulating factor; rhIL-4: recombinant human interleukin-4; ELISA: Enzyme-linked immunosorbent assay; PBMCs: Peripheral blood mononuclear cells; DCF: Dichlorodihydrofluorescein; p38 MAPK: p38 mitogen-activated protein kinase; IL: Interleukin; TNF-α: Tumour necrosis factor-α; DIC: Differential interference contrast; UAP: Unstable angina pectoris.

## Competing interests

The authors declare that they have no conflicts of interest.

## Authors’ contributions

HL, KY, DH, AJS, YNZ, JYQ and JBG were deeply involved in the conception and design of the study. KY and DH were responsible for the analyses of the data. HL drafted the manuscript. All authors read and approved the final manuscript.

## Authors’ information

HL, KY, DH, AJS, YZZ, JYQ, and JBG are members of the Department of Cardiology, Zhongshan Hospital, Fudan University, Shanghai Institute of Cardiovascular Diseases, 180 Fenglin Road, Shanghai China.
